# Neonatal factors associated with immediate low Apgar score in newborn babies in an intermediate hospital in Namibia: a case control study

**DOI:** 10.4314/ahs.v23i3.18

**Published:** 2023-09

**Authors:** Justina Lungameni, Emma Maano Nghitanwa, Laura Uusiku

**Affiliations:** University of Namibia, School of Nursing and Public Health

**Keywords:** Neonatal, factors, immediate low Apgar score, newborn, babies

## Abstract

**Introduction:**

Apgar score is conducted to a baby immediately after birth checking how the baby tolerated the birth process and outside the uterus.

**Objectives:**

To describe the neonatal factors associated with immediate low Apgar score and analysing the associations among factors associated with low Apgar score in new-born babies.

**Methods:**

A quantitative, case-control, descriptive research design was used. Study population were all maternal records of deliveries conducted between 01 January 2019 and 31 December 2019. Simple random sampling was used to select the sample size for 194 cases and 194 controls using a 1:1 case-control ratio. Records indicating low Apgar scores were the cases while normal Apgar scores were the controls. A total of 388 maternal files were reviewed. Data were collected using a document review checklist and analysed using SPSS version 26.

**Results:**

The study found that, neonatal factors associated with immediate low Apgar score are; gestational age, foetal presentation, cord prolapse, cord around the neck and the importance of cardiotocography interpretation as they had a P-value > 0.005.

**Conclusion:**

Gestational age, birth weight, foetal presentation, cord around the neck and lack of cardiotocography assessment were found to be associated with immediate low Apgar score.

## Introduction

Birth Asphyxia is a common neonatal problem globally. Birth asphyxia, defined as the failure to establish breathing at birth 1. Therefore, Apgar score is conducted on every newborn immediately after birth and at five minutes to check if the baby cope with birth process and how the newborn can adapt on the environment outside the uterus^1^. Apgar score was originally developed by Virginia Apgar, the anesthesiologist in America in 1953^2^. Good Apgar score of 7, 8, 9 and 10 assigned at one minute and five minutes of life indicates that the baby is healthy, while any score below 7 at one minute and five minutes of life and then at five-minute intervals until twenty minutes of life, is considered as low Apgar score 3. Tharpe, Farley and Jordan 4 indicated that new-born infants should make the transition from the intra-uterine environment, where nutritional and respiratory needs are met through the umbilical cord to the outside environment, where the infant must initiate breathing and suckling to survive. Most new-born babies delivered by midwives begin to breath spontaneously, while some need assistance to successfully make the transition to life outside the womb. Thus, Apgar score is used as a guide for resuscitation which the baby may require^3^.

In addition, 2.6 million neonates died in 2016 globally as a result of asphyxia and the majority of these neonatal deaths were concentrated in the first day and week of the child's birth, with about 1 million having died on the first day and close to one million dying within the next six days 5. In Africa, some countries have also experienced babies born with low Apgar scores. About 8% to 38% of babies delivered in West Africa may have low Apgar scores and over two-thirds of babies with low Apgar scores may result in perinatal mortality^6^.

A total of 27 (11.7%) of birth asphyxia was recorded in Namibia and 26% of birth asphyxia recorded in 2013 7 8. Birth asphyxia occurred when the baby is born with severe low Apgar score. A study conducted in Namibia in 2018, shows that 24% of the new-born babies had an Apgar score of between 0-2 at one minute and 7% of new-born babies had an Apgar score between 0-2 Apgar score at five minutes, 29% and 17% of new-born babies had an Apgar score between 3 - 4 at one minute and at five minutes respectively, while 47% and 76% of the newborn had an Apgar score between 5 - 7 respectively 9.

In addition, the 2018 and 2019 annual statistics for maternity ward in Onandjokwe Intermediate Hospital, recorded a total of 39 neonatal deaths. It has been noted that 11% of these neonatal deaths occurred as a result of birth asphyxia and these new-borns were recorded with low Apgar score at birth. Apgar score of less than 7 is associated with high risk of neonatal mortality and morbidity 10. Moreover, in 2018, a total of 6955 deliveries were conducted at Onandjokwe Intermediate Hospital of which 5.2% (366) new-borns had a low Apgar score^11^. Whereas, in 2019, annual statistics showed that deliveries conducted in Onandjokwe Intermediate hospital were 6954 of which 5.4% (376) new-born babies were rated with low Apgar score 12.

In addition, statistics of neonatal deaths reported in Onandjokwe Hospital due to low Apgar in 2018 were 16 (41%) and increased to 23 deaths (58.9%) in 2019. Therefore, it is important to assess the health status of the neonate during the birth process using validated methods such as the Apgar scoring system which is done in the first and every fifth minute respectively after birth.

The objective of the study was to describe the neonatal factors associated with immediate low Apgar score among new-born babies in Onandjokwe Intermediate Hospital.

## Methods

### Study design

A quantitative research approach was adopted using a descriptive, analytical, case -control research design.

### Setting

The study was conducted at Onandjokwe Intermediate Hospital, a 450-bed district hospital located in northwest Namibia, about 750km from the capital city Windhoek. It serves as the referral hospital for many district health facilities in the Ohangwena, Oshikoto and Oshana regions of Namibia and provides antenatal care, labour, neonatal intensive care and postnatal care services. Due to its geographical location and referral status, most women deliver their babies at this hospital. The hospital recorded an increase of babies born with low Apgar scores from 5.2 % in 2018 to 5.4% in 2019. In addition, according to the annual statistics reports of 2018 and 2019 for the maternity ward at Onandjokwe Intermediate Hospital, a total of 39 neonatal deaths were recorded.

### Population and Sampling

The population were all mothers with maternity records for deliveries conducted at Onandjokwe Intermediate Hospital from 01 January 2019 to 31 December 2019. Therefore, the population includes all mothers for 6954 maternity records for the deliveries conducted in 2019 regardless of the mode of delivery. The target population was all mothers 376 for maternity records with low Apgar score newborn babies and those with normal Apgar score from 01 January to 31 December 2019 regardless of the mode of delivery. Simple random sampling method was employed to select participants to the study.

The sample size was determined by using Yamane's Formula (n =_N_______1+N(a)2). at a 5% margin of error, whilst 194 controls were selected using a 1:1 case-control ratio. In this formula, ‘n’ represents the sample size, ‘N’ represents the population size under the study and ‘a represents the margin of error. The researcher used a 95% confidence level and 5% or 0.05 as the margin of error. Participants were selected from a table of random of maternal record up to the desired sample size of (194) of each category to the ratio of 1:1, namely new born babies with low and normal Apgar score. Each random number was used to select the corresponding record from the storage area until the sample size was achieved. All maternal records for the year 2019 had a chance of being selected for the study. The total sample size was thus 388 maternal records.

The inclusion criteria were all maternity records of mothers regardless of delivery type. All maternal files indicating home deliveries and those that were referred from other facilities after deliveries were excluded. Bias was minimized by selecting control group (maternal files with normal Apgar score) as a representative sample of the population which produced the cases (maternal files for low Apgar score.

### Data collection and analysis

Data was collected from June 2020 to August 2020 using a document review checklist developed by the researchers in English. The checklist consisted of two sections that collected data on socio-demographic characteristics and neonatal factors. Data were collected from 388 maternal record files consisting of 194 cases and 194 controls for the period between 01 January 2019 to 31 December 2019. The selected records were retrieved from the hospital storeroom, reviewed for data extraction and re-filed by the researchers. These maternity files were reviewed using a data collection tool (document review checklist) to indicate whether the attribute being measured was present or not. Validity and reliability of the data collection tool were ensured by getting input on the data collection tool from an obstetrician regarding clarity, relevance and simplicity of the content. Reliability was ensured by piloting the data collection tool on 10% of maternal records to ensure that all important variables of concern were covered.

Data was entered and cleaned and analysed using the Statistical Package for the Social Sciences (SPSS) version 26. Furthermore, the Chi-square test was used to measure the association between dependent variable (low Apgar score) and independent variables (factors)). In addition, odds (95% CI) were calculated as a measure of association and the Dependent variables were analysed using Binary Logistic regression in order to measure changes in the independent variables that were associated with changes in the probability. The level of significance was determined by factors with the p value less than 0.05% at 95% confidence interval.

### Ethical Consideration

Ethical approval was obtained from the University of Namibia Research Ethics Committee with protocol reference: SON N2020. Ethical principles of confidentiality and anonymity were maintained. Since maternal records were used as participants the principle of informed consent was not applicable, however permission to conduct the study was sought and obtained from Onandjokwe Intermediate Hospital management.

## Results

### Demographic profile of participants

As displayed in table 1 the demographic profile shows a great number of 227(59%) of the participants to be between 21 to 34 years and fewer participants constituting 77(20%) were less than 20 years old. About half of the participants were multigravida 191 (49%) followed by grand multigravida 73(19%). Furthermore, 148(38%) participants were multiparous followed by grand multiparas 36(9%).

**Table 1:** Demographic profile of participants

Variables	Frequencies	Percentages %
**Age Group of mothers**		
Less than 20	77	20%
21- 34	227	58%
35 an above	84	22°%
**Gravidity:**		
Primigravida	124	32%
Multigravida	191	49%
Grandmultigravida	73	19 %
**Parity:**		
Primiparous	71	18.3%
Multiparous	148	38.2%
Grand multiparous	36	9.2%
Nuliparous	133	34.3%

### Socio-demographic characteristics and association with immediate low Apgar score

The mean age for the whole sample was 29 years with a minimum of 14 years and a maximum of 36 years. Majority of mothers delivered low Apgar score babies, 106 (47%) were aged 21 -34 years, while 38 (49%) mothers were below 20 years. There was no statistically significant association between the mothers' age and immediate low Apgar score (p>0.897). The study shows that the highest number of mothers were multigravida for both low Apgar scores, 81 (43%) and normal Apgar scores, 109 (57%). Grand multigravida was below 50 in both low Apgar scores, 32 (44%), and normal Apgar score groups 40 (56%). Most mothers were nulliparous, 75 (57%), in the low Apgar score and 57 (43%), in the normal Apgar score group. In the low Apgar score group, only 68 (48%) were recorded as multiparous and 75 (52%) in the normal Apgar score. The immediate low Apgar score had statistically significant association with gravidity (p<0.021) and parity (p<0.029).

### Sex, gestational age and birth weight and association with low Apgar score

Male, low Apgar group had 91 (47.2%) babies and the normal Apgar score group had 102 (52.8%). In addition, the female low Apgar score group recorded 94 (48.2%) while the normal Apgar score group had 101 (51.8%). There was no statistically significant association found between the sex of the new-born baby and the immediate low Apgar scores (p>0.835). Regarding the gestational age of the new-born baby, majority320 (82, 5%), of newborns were delivered at full term. In contrast, newborn delivered at preterm were 59 (15, 2 %), small for dates 4(1%) and post-term deliveries were 2(0.5 %). However, 3 (0,8%) maternity records were incomplete and had missing data on gestational age.

Table 2 displays the associations between gestational age and immediate low Apgar score. Most of new-born babies delivered at full term, 136 (42.5%) had low Apgar while184 (57.5%) had normal Apgar scores immediately after delivery. Most 41 (69.5%) of the pre-term new-born babies had an immediate low Apgar score compared to 18 (30.5%) that had normal Apgar score. Small for dates and post-term deliveries had few newborns in both low and normal Apgar score groups. The study found a statistically significant association between immediate low Apgar score and gestational age (p<0.000). Furthermore, majority of new-born babies 293 (75%) were delivered with normal birth weight, comparing to low birthweight of 37 (10%), severe prematurity 31 (7%). Macrosomia was less represented at 24 (6%) and 3 (1) were not recorded and had missing data on birth weight. There was a statistically significant association between immediate low Apgar score and birth weight (p<0.000) as displayed in table 3.

**Table 2:** Association between gestational age and immediate low Apgar score

Variables	Low Apgar score (Cases)	Normal Apgar score (Control)	Total	P-value
Gestational age				0.000
Full-term	136 (42.5%)	184 (57.5%)	100	
Small for dates (GA)	3 (75.0%)	1 (25.0%)	100	
Post-term	2 (100.0%)	0 (0.0%)	100	
Pre-term	41 (69.5%)	18 (30.5%)	100	

**Table 3:** Association between birth weight and immediate Apgar score

Variables	Low Apgar Score (Cases)	Normal Apgar score (Control)	Total %	P-value
Birth weight				0.000
Severe premature (0.100kg1.900kg)	27 (87.1%)	4 (12.9%)	100	
Low birth weight (2.000kg-2.400kg)	22 (59.5%)	15 (40.5%)	100	
Normal birthweight (2.500kg3.900kg)	120 (41.0%	173 (59.0%	100	
Macrocosmic (>4.000kg)	13 (54.2%)	11 (45.8%)	100	

### Fetal complications and fetal presentation and association with immediate low Apgar score

Majority 379 (97.7%) of mothers that deliver new born with low Apgar score had no cord prolapse and only few 2 (0.5%) babies were diagnosed with congenital abnormalities and 10 (2.6%) were diagnosed with cord around the neck. Furthermore, all new-born babies born with cord prolapse 9 (100.0%) and congenital abnormality 2 (100.0%) had a low Apgar score. Of the new-born babies delivered with a cord around the neck 9 (90.0%) had a low Apgar score and 1 (10.0%) had a normal Apgar score. There was a significant statistical association between immediate low Apgar score and foetal presentation (p<0.008), cord prolapse (p<0.001) and cord around the neck (p<0.007), while congenital abnormality was not found statistically associated with the immediate low Apgar score as the (p>0.137). Furthermore, most of the new-born babies were delivered in vertex presentation had normal Apgar score 197 (54.6%) and the low Apgar score group had 164 (45.4%). The new-born babies delivered with the compound presentation 6 (83.6%) had a low Apgar score. There was a statistically significant association between foetal presentation and immediate low Apgar score (p<0.001).

### Cardiotocography (CTG) interpretation and association with immediate Low Apgar score

Table 4 shows that most of the new-born babies' CTG assessment were not printed and were more constituting the most in both, low Apgar score 128 (40.1%) and normal Apgar score groups 191 (59.9%). Acceleration reported a few neonates in low Apgar group 9(69.2%) while, in the normal Apgar score group the lowest percentage was reported in deceleration category 1 (6.7%). There was a statistically significant association found between immediate low Apgar score and Cardiotocography (p<0.000).

**Table 4:** Association between Cardiotocography assessment (CTG) and immediate Apgar score

Variables	Low Apgar score (Cases)	Normal Apgar score (Control)	Total%	P value
Cardiotocography				0.000
Deceleration	14 (93.3%)	1 (6.7%)	100	
Acceleration	9 (69.2%)	4 (30.8%)	100	
Non-reassuring	17 (89.5%)	2 (10.5%)	100	
Reassuring	17 (77.3%)	5 (22.7%)	100	
CTG not printed	128 (40.1%)	191 (59.9%)	100	

## Discussion

The findings in this study shows that maternal age groups had almost a similar percentage of new-born babies having low Apgar score immediately, even though it was not observed as an associate factor for immediate low Apgar score (p>0.897). Similarly, in Iran and Malaysia Tewesa *et al.* 13, 14 observed no association in maternal age groups less than 20 or above 35 years old. However, the study in Ethiopia found an association between maternal age group of 15-25 years old which is different from the findings of this study 15. Young mothers tended to have difficult deliveries due to uncooperative behaviours during the first and second stages of labour that caused distress to the foetus. In contrast to this study findings Abdo *et al.* 16 established that Ethiopian mothers aged >35 years were associated with low Apgar score than the young maternal age as they were at the risk of caesarean sections and inadequate pelvic.

The findings reveal association between Gravidity and Parity and immediate Apgar score in this study. Primigravida were highlighted to be a common factor in this study as they indicated a larger percentage of 58.3%. Likewise, in Malaysia and Ethiopia, studies show that gravidity and primigravida were associated factors to low Apgar score due to inadequate experience of pain in primigravida they tend to be uncooperative 13,17. Furthermore, nulliparous recorded 56.8% delivered low Apgar score in this study. Similarly,^14^ observed that in Iran previous live birth associated with high Odds of low Apgar score. While, in Tanzania and Nigeria, it was reported that grand multiparous and primiparity are at great risk of low Apgar score 3,18 ,19.

This current study recorded neonatal factors such as gestational age, birth weight, foetal presentation, cord around the neck and cardiotocography associated with immediate low Apgar score. Female and male new-born babies were almost equal in having low Apgar score in this study. Contrary, Omokhodion *et al.* 20 found that male new-born babies associated more with low Apgar score than females. This was due to the fact that they tended to be delivered by instrumental delivery as well as caesarean section for failure to progress in the normal way. Additionally, gestational age was an associate factor of low Apgar score in this study. The youngest gestational ages have a higher proportion of low Apgar score. This is because they tend to have preterm labour and delivered before pregnancy become term. Birth weight was also found to be associated with immediate low Apgar score in this study. Severe premature new-born baby's weight 0.100kg to 1.900kg made up the highest percentage in this study because of inability to breath spotaneously after delivery as they have immature lungs. Similarly, in Nigeria, Mohammed 21 found that low birth weight less than 2.5kg associated more with low Apgar score at one minute due to immaturity of organ and nervous system. On the contrary, a study by in Ethiopia and Nigeria, respectively, found that new-born babies with birth weight >4kg were associated with Cephalopelvic Disproportion (CPD) resulting in prolonged labour that cause low Apgar score and leads to complications of shoulder dystocia and causes injuries such as plexus brachialis 18, 22.

Moreover, foetal presentation (*p*<0.00), cord pro-lapse(*p<*0.000), cord around the neck (*p*<0.000) and Cardiotocography assessment (*p*<0.000) were found to be associated with low Apgar score in this study. Women in labour who had CTG with late deceleration, delivered new-born babies with low Apgar score compared to newborn babies due to decreased oxygen to the feotus. This is supported by Bogdanovic^23^ who maintains that the late deceleration associate with tachycardia and bradycardia can result in new-born delivered with low Apgar score.

## Limitations

The study used an adequate sample; however, the results could not be generalized because it was conducted in one hospital. The study was a retrospective study using documented data, s therefore there would be missing data and some inaccuracies in reporting. Large number of maternal record (388) were used in the study and that represented the majority of women who delivered in the intermediate hospital were the study was conducted.

## Conclusion

The findings of this study have shown different neonatal factors associated with immediate low Apgar score with p-value <0.000 such as, gestational age (p<0.000), foetal presentation (p<0.008), cord prolapse (p<0.001), cord around the neck (p<0.007), Lack of cardiotocography assessment (p<0.000). The study recommends that mid-wives should use existing policies and guidelines in monitoring women in labour for early identification of neonatal factors associated to low Apgar score. In addition, emphasis should be put on the strengthening plus the implementation of the recommendations by the World Health Organization (WHO)That will promote proper management, prevent complications and reduce the neonatal mortality rate in Namibia.

### What is already know on this topic

- Low Apgar score is increased at the study site
- Low Apgar score can lead to neonatal death

### What this study adds

- Neonatal factors contributing to immediate Apgar score identified.
